# Ocluded Anus

**Published:** 1868-04-15

**Authors:** H. A. Chase

**Affiliations:** Viroqua, Wisconsin


### OCCLUDED ANUS.

BY H. A. CHASE, M.D., VIROQUA, WISCONSIN.

In September, 1867, I was called to attend Mrs. A., in labor with her first child. After a somewhat protracted labor she was delivered of a male child. Five hours after completion of the labor I called and was informed by the nurse that the child had been vomiting for some time, and there had been no passage of the meconium, but in passing its urine it had passed dark specks per penis. Upon examination I found the anus absent, the raphe or median line extending as far back as the tip of the coccyx. I could detect no pouching of the intestine. Having decided to operate, I passed a straight narrow bladed bistoury in the direction of the rectum, but failed to establish an exit for the retained matter.

The child lived several days passing small quantities of meconium by the penis.

After death, permission having been granted, I made an examination and found the bladder distended with meconium.

The descending portion of the colon, instead of ending in the sigmoid flexure, ended in the bladder, with entire absence of rectum. All the other organs were well formed. The father informed me that when fourteen years of age he had an attack of diarrhœa that run along for several days, and then instead of passing the fæcal matter per anus, for three days he passed it all per penis, and had no stool the usual way during that time. He informed me the matter he passed had the same smell and look as fæces, and was passed in large quantities. At the end of three days he had regular stools every few hours, and his urine became clear.

